# De Senectute

**Published:** 1905-04

**Authors:** 


### DE SENECTUTE.

DR. WILLIAM OSLER has come into much newspaper notoriety over his unfortunate serio-comic expressions upon the age of effectiveness. It is too easy to multiply examples of men who have waited until old age began to grip them before achievement came. Every branch of human industry is full of old men who are steadily advancing on a rising gradient, combined achievement, and may prove their claim to exemption to the Lethean draught.

But taking men as they come, the olla podrida of mankind, Dr. Osler is nearer the truth than the paragraphers will admit.

The score of years that lie between twenty and forty are beyond quibble the cumulative ones. The period when the psychologically styled *tabula rasa* begins to receive the bulk of intellectual impressions. The mind is eminently susceptible at this time, and impressions to be stored up come thick and fast. It is also the age when in the lives of most men the heaviest strain falls—the strain that tests the metal and demonstrates the qualities that make for success, and the frailties that stand for failure.

The expression that at forty a man is either a fool or a doctor, makes a rather blunt point on the situation. It means that one has found his natural position by gravitation or "levitation." The problem then is to hold it against the field.

When a man lapses into old age the total of experiences that are to fall to him is nearly made up.

Whatever occurs is very apt to have fallen before in a manner so similar as to establish a precedent for action. He then finds himself threshing old straw—things are apt to be *rechauffé*. When one has reached this dead calm, this blasé state of tranquillity, completeness and sluggishness, the query is pertinent of his lagging superfluous on the stage.

The time will hardly come when the old of the tribe, after the former custom of some of the South Sea Islanders, are taken to the top of some lofty palm and politely, reverently but firmly requested to step down and off.

But barring this tree-climbing formality, the civilized aged virtually carries out the savage custom—not abruptly, but by insensibly yielding to pressure and the consciousness that the segments of the circle of life are nearly all in position. This is natural, graceful and fitting.

